# Impact of treatment and re-treatment with artemether-lumefantrine and artesunate-amodiaquine on selection of *Plasmodium falciparum* multidrug resistance gene-1 polymorphisms in the Democratic Republic of Congo and Uganda

**DOI:** 10.1371/journal.pone.0191922

**Published:** 2018-02-01

**Authors:** Vito Baraka, Hypolite Muhindo Mavoko, Carolyn Nabasumba, Filbert Francis, Pascal Lutumba, Michael Alifrangis, Jean-Pierre Van geertruyden

**Affiliations:** 1 National Institute for Medical Research, Tanga Centre, Tanga, United Republic of Tanzania; 2 Global Health Institute, University of Antwerp, Antwerp, Belgium; 3 Département de Médecine Tropicale, Faculté de Médecine, Université de Kinshasa, Kinshasa, Democratic Republic of Congo; 4 Epicentre Mbarara Research Base, Mbarara, Uganda; 5 Centre for Medical Parasitology at the Department of Immunology and Microbiology, University of Copenhagen, Copenhagen, Denmark; 6 Department of Infectious Diseases, National University Hospital (Rigshospitalet), Copenhagen, Denmark; George Washington University School of Medicine and Health Sciences, UNITED STATES

## Abstract

**Background:**

The emergence of resistance against artemisinin combination treatment is a major concern for malaria control. ACTs are recommended as the rescue treatment, however, there is limited evidence as to whether treatment and re-treatment with ACTs select for drug-resistant *P*. *falciparum* parasites. Thus, the purpose of the present study is to investigate the impact of (re-)treatment using artesunate-amodiaquine (ASAQ) and artemether-lumefantrine (AL) on the selection of *P*. *falciparum multidrug resistance-1* (*Pfmdr1*) alleles in clinical settings.

**Methods:**

*P*. *falciparum* positive samples were collected from children aged 12–59 months in a clinical trial in DR Congo and Uganda. *Pfmdr1* single nucleotide polymorphisms (SNPs) analysis at codons N86Y, Y184F, and D1246Y were performed at baseline and post-treatment with either AL or ASAQ as a rescue treatment using nested PCR followed by restriction fragment length polymorphism (RFLP) assays.

**Results:**

The pre-treatment prevalence of *Pfmdr1* N86 and D1246Y varied significantly between the sites, (p>0.001) and (p = 0.013), respectively. There was borderline significant directional selection for *Pfmdr1* 184F in recurrent malaria infections after treatment with AL in Uganda site (p = 0.05). *Pfmdr1 NFD* haplotype did not significantly change in post-treatment infections after re-treatment with either AL or ASAQ. Comparison between pre-treatment and post-treatment recurrences did not indicate directional selection of *Pfmdr1* N86, D1246 alleles in the pre-RCT, RCT and post-RCT phases in both AL and ASAQ treatment arms. *Pfmdr1* 86Y was significantly associated with reduced risk of AL treatment failure (RR = 0.34, 95% CI:0.11–1.05, p = 0.04) while no evidence for D1246 allele (RR = 1.02; 95% CI: 0.42–2.47, p = 1.0). Survival estimates showed that the *Pfmdr1* alleles had comparable mean-time to PCR-corrected recrudescence and new infections in both AL and ASAQ treatment arms.

**Conclusion:**

We found limited impact of (re-)treatment with AL or ASAQ on selection for *Pfmdr1* variants and haplotypes associated with resistance to partner drugs. These findings further supplement the evidence use of same or alternative ACTs as a rescue therapy for recurrent *P*.*falciparum* infections. Continued monitoring of genetic signatures of resistance is warranted to timely inform malaria (re-)treatment policies and guidelines.

## Introduction

Global malaria control efforts have expanded significantly. However, malaria remains a major health challenge with approximately 212 million new cases and 429,000 malaria-related mortality in 2015[1]. The success is mainly attributed to scaling up of coverage with insecticide-treated nets (ITNs) and deploying artemisinin-based combination therapies (ACTs) for the treatment of uncomplicated *Plasmodium falciparum* malaria [2]. The efficacy of ACTs depends on the synergistic action of short-acting artemisinin derivatives and a longer acting partner drug that eliminates the residual parasite load[3]. However, recent reports have documented diminishing efficacy of ACTs in Great Mekong Sub-region (GMS), raising serious concerns regarding the future of malaria control and elimination [4–6]. Despite these reports, ACTs remain largely efficacious with adequate cure rates in sub-Saharan Africa (SSA) [7,8]. In order to maintain ACTs as the cornerstone for malaria treatment, it is crucial that the artemisinin derivatives and their longer acting partner drugs retain high efficacy. Currently, the most used partner drugs in SSA are amodiaquine and lumefantrine which are combined with artesunate (artesunate-amodiaquine, ASAQ) or artemether (artemether-lumefantrine, AL).

Treatment failure is a complex problem interplay of several factors ranging sub-optimal drugs exposure, poor adherence to treatment, host-genetics factors and/or the emergence of drug resistance. Recrudescence rates are low in most countries in Africa and ACTs retain high efficacy as the first-line therapy for malaria treatment, the few reported recurrent infections are commonly new infections [7,8]. ACTs are increasingly an integral part of malaria control and are the recommended “rescue treatment” or “second-line treatment” for the treatment of recurrent *P*.*falciparum* infections in addition to Quinine+antibiotic [9]. However, the emergence of resistance/tolerance against the long-acting partner drugs, such as amodiaquine and lumefantrine, may consequently affect the efficacy of the ACTs and increase the odds for emerging resistance to the short-acting artemisinin-derivatives. Monitoring is, therefore, crucial to understanding the development of a drug-mediated selection of *Pfmdr1* alleles in the patients that are treated and re-treated with ACTs as a rescue treatment. In the Democratic Republic of Congo, uncomplicated malaria cases are treated with ASAQ as the first line while in Uganda, AL is the recommended regimen.

Amodiaquine (AQ) is a slow-acting 4-aminoquinoline antimalarial drug with a terminal plasma half-life between 9–18 days, acting through its active metabolite desmethyl amodiaquine [10]. Lumefantrine is an arylamino-alcohol drug with a half-life of 3–5 days and has shared structural similarity to mefloquine and quinine[10]. The molecular targets and mechanisms of action of lumefantrine are yet to be fully unravelled [11]. However, deteriorating clinical outcomes of various antimalarial drugs, including some of the partner drugs, in particular to 4-aminoquinoline such as chloroquine (CQ), amodiaquine (AQ) and piperaquine (PQ) and aryl-amino alcohol based drugs (lumefantrine, quinine and mefloquine) is alarming as may increase rates of treatment failures.

*P*. *falciparum multidrug resistance transporter 1* (*Pfmdr1*) gene occurs on Chromosome 5 and encodes for *P-glycoprotein homologue 1* (*Pgh1*) protein located in the digestive vacuole of the parasite. *Pfmdr1* is of interest because of the described role of the polymorphisms at codon N86Y, Y184F, S1034C, N1042D, and D1246Y in drug resistance. The polymorphisms were associated with altered parasite susceptibility to several classes of antimalarials [11–15]. Drug pressure due to artemisinin derivatives partner drugs was shown to exert directional selection of *Pfmdr1* 86Y, Y184 and 1246Y variants in amodiaquine(AQ) and while AL and Mefloquine (MQ) inversely selected for N86, 184F D1246 variants [12,16–18]. Similar observations were documented in *in vitro* and *ex vivo* susceptibility assays [19–21]. *Pfmdr1* SNPs exert altered susceptibility profile to other antimalarials including mefloquine(MQ), chloroquine(CQ) and quinine(QN) [13,14]. Interestingly, it was recently shown that *Pfmdr1* 86Y increases parasite susceptibility to dihydroartemisinin, which is the active artemisinin metabolite of artemisinin derivatives[15].

In addition, *Pfmdr1* haplotypes are an important determinant in modulating the level of resistance. Early treatment failures(ETF) that occur between day 2–3 post-treatment with AL and DHA-PQ, were more significantly shown to harbour *Pfmdr1 NFD* haplotype in the previous study [22]. Furthermore, *Pfmdr1* haplotypes are differentially selected, for instance, *Pfmdr*1 *YYY* haplotype (86Y, Y184 and 1246Y) was shown to be selected after exposure to artesunate-amodiaquine(ASAQ) while AL inversely exerted selective pressure of *Pfmdr1*-*NFD* haplotype(N86, 184F and D1246) [16,23]. The observation was further exemplified by Malmberg *et al* [24] that well demonstrated the ability of the parasites harbouring *Pfmdr1 NFD* haplotype to withstand a 15-folds concentration of lumefantrine compared to *Pfmdr1 YYY* in re-infecting parasites [24]. Also, evidence suggests an association between the *Pfmdr1-NFD* and increased risk of gametocyte carriage following treatment with AL [23]. In addition, variation in *Pfmdr1* copy number (CNVs) has been shown to modulate drug sensitivity pattern of the partner drugs, mefloquine, lumefantrine and piperaquine, confined in the SEA in the Great Mekong sub-region [12,25,26].

We investigated the impact of subsequent treatment courses with ACTs, either the same or alternative, on the selection of *Pfmdr1* polymorphisms in clinical settings in DR Congo and Uganda.

## Material and methods

### Study design and participants

The protocol and the outcome of the clinical trial for this molecular analysis were published separately [27,28]. Briefly, the trial was a bi-centre, open-label, randomised, three-arm phase 3 trial (2:2:1 ratio) conducted in Lisungi health centre in DR Congo, and Kazo health centre in Uganda in 2012–2014. In the pre-randomized controlled (Pre-RCT) phase of the trial, the patients were treated with the first-line treatment as per respective country i.e. artesunate-amodiaquine (ASAQ) in DR Congo and artemether-lumefantrine (AL) in Uganda and followed up for 42 days. Patients who experienced malaria between day 14–42 were enrolled in randomised control trial (RCT) phase and assigned either ASAQ, AL or Quinine+clindamycin(QnC). The patients that presented with clinical or parasitological failure post day 14 were recruited in post-randomization control (post-RCT) trial phase and were re-treated according to the country malaria treatment guideline [28].

### Sample collection

*P*. *falciparum* microscopically positive samples for this study were collected in Uganda and DR Congo as part of the QuinACT clinical trial described above [28]. We analysed samples in pre-RCT phase, RCT phase and post-RCT phases from ASAQ and AL arms of the QuinACT trial. The samples from QnC arm were excluded because *Pfmdr1* is not associated with Quinine resistance. Fingerprick samples of blood were collected on filter paper (Whatman 3MM, Maidstone, UK), air-dried, labelled and stored in a desiccator containing silica gel until further processing.

### DNA extraction

DNA isolation from dried blood spots was done using QIAmp DNA Mini Kit as per manufacturer’s instructions (Qiagen^®^, Hilden, Germany). Isolated DNA was stored at -20°C until further use in the downstream applications.

### PCR and RFLP assays

The extracted samples were amplified by outer and nested PCR protocols for amplification of *Pfmdr1* genes targeting single nucleotide polymorphisms (SNPs) at codons N86Y, Y184F and D1246Y followed by restriction fragment length polymorphism (RFLP) digestion using restriction enzymes as previously described [29].

### Data analysis

Data management and analyses were performed using Stata, version 13(StataCorp, College Station, TX, USA). Prevalence of *Pfmdr1* N86Y, Y184F and D1246Y alleles and haplotypes were calculated and compared between the treatment arms and sites. Mixed alleles are presented in prevalence data, however, excluded when constructing the *Pfmdr1* haplotypes. Chi-square or Fisher exact tests were used to assess the associations between categorical variables. McNemar’s test or exact McNemar's test were used to determine the directional selection for *Pfmdr1* alleles in patients with *P*.*falciparum* recurrent infections in matched analysis between pre-treatment and post-treatment in the different trial phases. Risk Ratios (RR) and adjusted odds ratios (aORs) with 95% confidence intervals (CIs) were used to assess the association between *Pfmdr1* SNPs or haplotypes and treatment outcomes. Multivariate logistic regression was used to determine factors associated with PCR-corrected treatment failures and new infections adjusted for such as age, site, anaemia, fever, and parasite density. The cumulative risk of treatment failure or new-infections by *Pfmdr1* alleles and haplotypes were analysed using Kaplan-Meier survival analysis and the comparison was made by using log-rank test. *P*-values were considered significant at ≤0.05.

### Ethical considerations

The study was approved by the Ethical Committee of the University of Antwerp, Belgium, the Uganda National Council for Science and Technology, Uganda and the Ethics Committee of the School of Public Health, University of Kinshasa, DR Congo. Informed consent was obtained from all participants through their parents or legal guardians [27]

## Results

### Demographic and clinical characteristics of patients

A total of 755 samples were analysed for *Pfmdr1* SNPs polymorphisms in pre-RCT, RCT and post-RCT phases. Of those, proportions of the successfully genotyped samples were; 732/755 (97%), 729/755 (96.6%) and 736/755 (97.5%) in codon 86, 184 and 1246 respectively. Of those, a total of 242 patients with uncomplicated *P*.*falciparum* infections were randomised to either ASAQ (n = 114) or AL (n = 128) (Table 1). The proportion of males and females enrolled was similar between both study sites and also by treatment randomization (Table 1). The mean weight was 12.7kg and 12.0kg in DR Congo and Uganda, respectively and was also similar between treatment arms. In terms of the clinical characteristics, the geometric mean parasitemia was comparable by site and treatment allocation. The proportion of the treatment allocation for patients receiving AL *vs* ASAQ was 56.5% *vs* 43.5% and 50% *vs* 50% in Congo and Uganda, respectively. The bednet ownership was approximately 50% for each site and was marginally higher in AL (58.7) *vs* ASAQ (41.3) treatment arm, (p = 0.07), (Table 1).

**Table 1:** Patients demographic and clinical characteristics at baseline of the randomisation phase of the QuinACT trial in DR Congo and Uganda sites.

Characteristics	Study sites			Treatment allocation	
	Total (n = 242)	DR Congo (n = 108)	Uganda (n = 134)	AL(n = 128)	ASAQ(n = 114)
Number (%) male	129(53.3)	60 (46.5)	69(53.5)	68(50.9)	61(53.5)
Weight (kg) (median [IQR])	12.5(9.0–20.5)	12.7(9.0–18.0)	12.0(9.0–20.5)	12.5(9.0–20.5)	12.35(9.0–19.0)
Age (months) (median[IQR])	34(25.0–48.0)	34(26.5–49.5)	34.0 (24–44)	34.5(24.5–48.0)	34.0(25.0–44.0)
No. of patients in age groups:					
<23 months^¥^	53(21.9)	21(19.4)	32.0(23.9)	31.0(58.5)	22.0(41.5)
24–35 months	79(32.6)	37(34.5)	42.0(31.3)	37.0(46.8)	42.0(53.2)
36–47 months	49(20.3)	17(15.7)	32.0(23.9)	25.0(51.0)	24.0(49.0)
48–60 months†	61(25.2)	33(30.6)	28.0(20.9)	35.0(57.4)	26.0(42.6)
Temperature (°C) (median [IQR])	37.8(36.1–41.8)	38.1 (36.2–41.3)	37.8(36.1–41.8)	37.9(36.2–41.8)	37.8(36.1–41.4)
Treatment allocation					
Artemether Lumefantrine (AL) n (%)	128.0(52.9)	61(56.5)	67.0(50.0)	-	-
Artesunate Amodiaquine (ASAQ) n (%)	114.0(47.1)	47(43.5)	67.0(50.0)	-	-
Bednet ownership^□^, n (%)	121.0(50.0)	60.0(49.6)	61.0(50.4)	71.0(58.7)	50.0(41.3)
Haemoglobin in g/dL, (range)	10.3(6.0–14.4)	10.4(6.2–14.4)	10.2(6.0–14.1)	10.4(6.0–14.4)	10.3(6.5–14.1)
GMP density (95% CI)	32137(26588–38843)	34997(27361–44765)	30003(22648–39748)	33200(25270–43617)	30984(23773–40385)

IQR = Interquartile ranges, AL = artemether-lumefantrine, ASAQ = artesunate-amodiaquine, GMP = geometric mean parasite density. The characteristics were comparable, except; Bednet ownership (p = 0.007)^□^ and Number of patient in age group (48-60months)^†^ and (<23 months)^¥^ as stratified per treatment allocation, (p = 0.02) and (p = 0.008), respectively, were statistically different.

### Baseline prevalence of *Pfmdr1* alleles and combined *Pfmdr1* haplotypes by the site during the RCT phase

Overall, in DR Congo the *Pfmdr1* SNPs results revealed that about half of the parasites harboured *Pfmdr1* N86, whereas in Uganda it was high almost at fixation point(52.8% *vs* 91%), (χ^2^ = 38.26, p<0.001). The mutant *Pfmdr1* 86Y was found at high proportion in DR Congo in comparison to Uganda (37.9% *vs* 7.5%). *Pfmdr1* Y184 (wildtype) variant was observed at high proportions at both sites (56.5% *vs* 67.2%), (χ^2^ = 5.04, p = 0.08). The prevalence of *Pfmdr1* D1246 was significantly higher in DR Congo in comparison to the Ugandan site (88.9% *vs* 73.9%), (χ^2^ = 8.65, p = 0.013). Logically, the Ugandan site harboured higher prevalence of the *Pfmdr1* 1246Y (9.2% *vs* 20.9%). The prevalence of the *Pfmdr1*-*NFD* and non-*NFD* haplotypes did not seem to vary between the sites (χ^2^ = 0.06, p = 0.81), (Table 2).

**Table 2:** Baseline prevalence of *Pfmdr1* polymorphisms and haplotypes among isolates from DR Congo and Uganda site in the randomisation phase.

SNPs codon	*Pfmdr1* variants	Baseline prevalence at RCT				*P*-value
		DR Congo (n = 108)		Uganda (n = 134)		
		n/N	% [95%, CI]	n/N	% [95%, CI]	
c86	N86 (Wildtype)	57/108	52.8[43.4–62.2]	122/134	91.0[86.2–95.9]	
	N86Y(Mixed)	10/108	9.3[3.8–14.7]	2/134	1.5[0–3.5]	<**0.001**
	86Y (Mutant)	41/108	37.9[28.8–47.1]	10/134	7.5[3.0–11.9]	
c184	Y184(Wildtype)	61/108	56.5[47.1–65.8]	90/134	67.2[59.2–75.1]	
	Y184F(Mixed)	3/108	2.8[0–5.9]	7/134	5.2[1.5–9.0]	0.08
	184F (Mutant)	44/108	40.7[31.5–50.0]	37/134	27.6[20.0–35.2]	
c1246	D1246 (Wildtype)	96/108	88.9[83.0–94.8]	99/134	73.9[66.4–81.3]	
	D1246Y (Mixed)	2/108	1.9[0–4.4]	7/134	5.2[1.5–9.0]	**0.01**
	1246Y (Mutant)	10/108	9.2[3.8–14.7]	28/134	20.9[14.0–27.8]	
c86-c184-c1246	*NFD* haplotype^†^	18/62	29.0[17.7–40.3]	29/94	30.9[21.5–40.2]	0.81
	*Non-NFD* haplotype^†^	44/62	71.0[59.7–82.3]	65/94	69.1[59.8–78.5]	

RCT = randomisation phase of the clinical trial; *Pfmdr1* = *P*.*falciparum multidrug resistance 1 gene*; SNPs = Single nucleotide polymorphisms; n = sample size; *NFD* = Combination *N86–184F-D1246* combination.

^†^Haplotypes excluding mixed alleles.

*P*-values are based on the Pearson's Chi-square test. Significant values are presented in bold type.

### Selection of *Pfmdr1* alleles and haplotypes associated with ASAQ and AL resistance in different trial phases

The analyses of the matched samples at baseline (D0) in comparison to the post-treatment recurrence infections (PCR-corrected recrudescent and new-infections) at the pre-RCT, RCT and post-RCT phases were performed using McNemar’s test for isolates at *Pfmdr1* at codon 86, 184, and 1246 (Table 3). Following treatment with ASAQ in DR Congo in the pre-RCT, there was no evidence to suggest directional selection for *Pfmdr1* 86Y (p = 0.32), Y184 (p = 0.13) and 1246Y (p = 0.27) alleles in the recurrent infections. Likewise, in the RCT phase where the patients were re-treated with the same treatment (ASAQ) or the alternative treatment (AL), there was no apparent selection of certain *Pfmdr1* alleles, albeit the sample size was low. Similar results were found for the post-RCT phase.

**Table 3:** Drug-mediated directional selection of the *Pfmdr1* alleles at codon 86, 184, and 1246 in isolates collected from children with recurrent malaria infections following (re-)treatment with AL or ASAQ.

Phase	*Pfmdr1* variants	Genotype change	ASAQ treatment arm		AL treatment arm	
			Discordant allele at recurrence compared to baseline (Dx/D0 (%))	MacNemar *χ*^*2*^(*P*-value)	Discordant allele at recurrence compared to baseline (Dx/D0 (%))	MacNemar *χ*^*2*^ (*P*-value)
Pre-RCT^‡^	*Pfmdr1* N86	N→Y	28/59(47.5)	1.0(0.32)	10/109(9.2)	2.79(0.07)
	*Pfmdr1* 86Y	Y→N	21/31(67.7)		19/20(95.0)	
	*Pfmdr1* F184	F→Y	13/32(40.6)	2.3(0.13)	22/99(22.2)	3.67(0.055)
	*Pfmdr1* 184Y	Y→F	22/58(37.9)		11/25(44.0)	
	*Pfmdr1* D1246	D→Y	9/97(9.3)	1.92(0.17)	15/94(16.0)	0.27(0.6)
	*Pfmdr1* 1246Y	Y→D	4/5(80.0)		18/26(69.2)	
RCT^a^	*Pfmdr1* N86	N→Y	1/11(9.1)	1.0^†^	0/13(0)	0.25^†^
	*Pfmdr1* 86Y	Y→N	1/2(50.0)		3/3(100.0)	
	*Pfmdr1* F184	F→Y	3/4(75.0)	1.0^†^	1/3(33.3)	1.0^†^
	*Pfmdr1* 184Y	Y→F	2/11(18.2)		1/11(9.1)	
	*Pfmdr1* D1246	D→Y	4/11(36.4)	0.37^†^	2/12(16.7)	1.0^†^
	*Pfmdr1* 1246Y	Y→D	1/3(33.3)		2/3(66.7)	
RCT^b^	*Pfmdr1* N86	N→Y	1/3(33.3)	0.63^†^	1/10(10.0)	1.0^†^
	*Pfmdr1* 86Y	Y→N	3/3(100.0)		0/4(0)	
	*Pfmdr1* F184	F→Y	1/3(33.3)	0.50^†^	0/3(0)	1.0^†^
	*Pfmdr1* 184Y	Y→F	0/4(0)		0/10(0)	
	*Pfmdr1* D1246	D→Y	0/7(0)	1.0^†^	0/15(0)	1.0^†^
	*Pfmdr1* 1246Y	Y→D	0(0)		0(0)	
Post-RCT^‡^	*Pfmdr1* N86	N→Y	1/1(100.0)	1.0^†^	1/24(4.2)	0.38^†^
	*Pfmdr1* 86Y	Y→N	2/3(0.67)		4/5(80.0)	
	*Pfmdr1* F184	F→Y	0/1(0)	1.0^†^	1/1(100.0)	1.0^†^
	*Pfmdr1* 184Y	Y→F	0/2(0)		1/1(100.0)	
	*Pfmdr1* D1246	D→Y	1/4(25.0)	1.0^†^	1/2(50.0)	1.0^†^
	*Pfmdr1* 1246Y	Y→D	0(0)		0(0)	

ASAQ = Artesunate-amodiaquine; AL = artemether-lumefantrine;

^‡^ = Treatment were administered according to the country recommended guideline i.e AL in Uganda and ASAQ in DR Congo; Pre-RCT = pre-randomized controlled trial phase; RCT = randomized controlled trial phase; Post-RCT = post-randomized controlled trial phase;

^a^ = Uganda site;

^b^ = DR Congo site;

n = number of samples at baseline; Dx = *Pfmdr1* allele changed in the recurrence infections (all PCR-corrected recrudescent and new-infections);

^†^ = Exact McNemar's test was used if n<5. The directional selection of the marker was tested by using McNemar’s *χ*^*2*^-test or Exact McNemar's test for paired pre- and post-treatment samples. Mixed alleles were excluded from the analysis. Significant values are presented in bold type.

Similarly, in Ugandan site, no significant evidence for directional selection of *Pfmdr*1 N86 (p = 0.07) and D1246 (p = 0.6) in patients with recurrent infections treated with AL treatment in the pre-RCT phase. However, there was there was marginal evidence to suggest a directional selection of *Pfmdr1* 184F (p = 0.0555), (Table 3). For the RCT phase, patients who received same AL as a rescue treatment and those received alternative treatment, ASAQ, no significant selection for the *Pfmdr1* allele was detected, (Table 3). Also, similar findings were observed in post-RCT where patients with recurrent infections and treated using AL. However, the sample size was low to provide statistical power for the recurrent infections in the RCT and Post-RCT phase.

**Fig 1:**
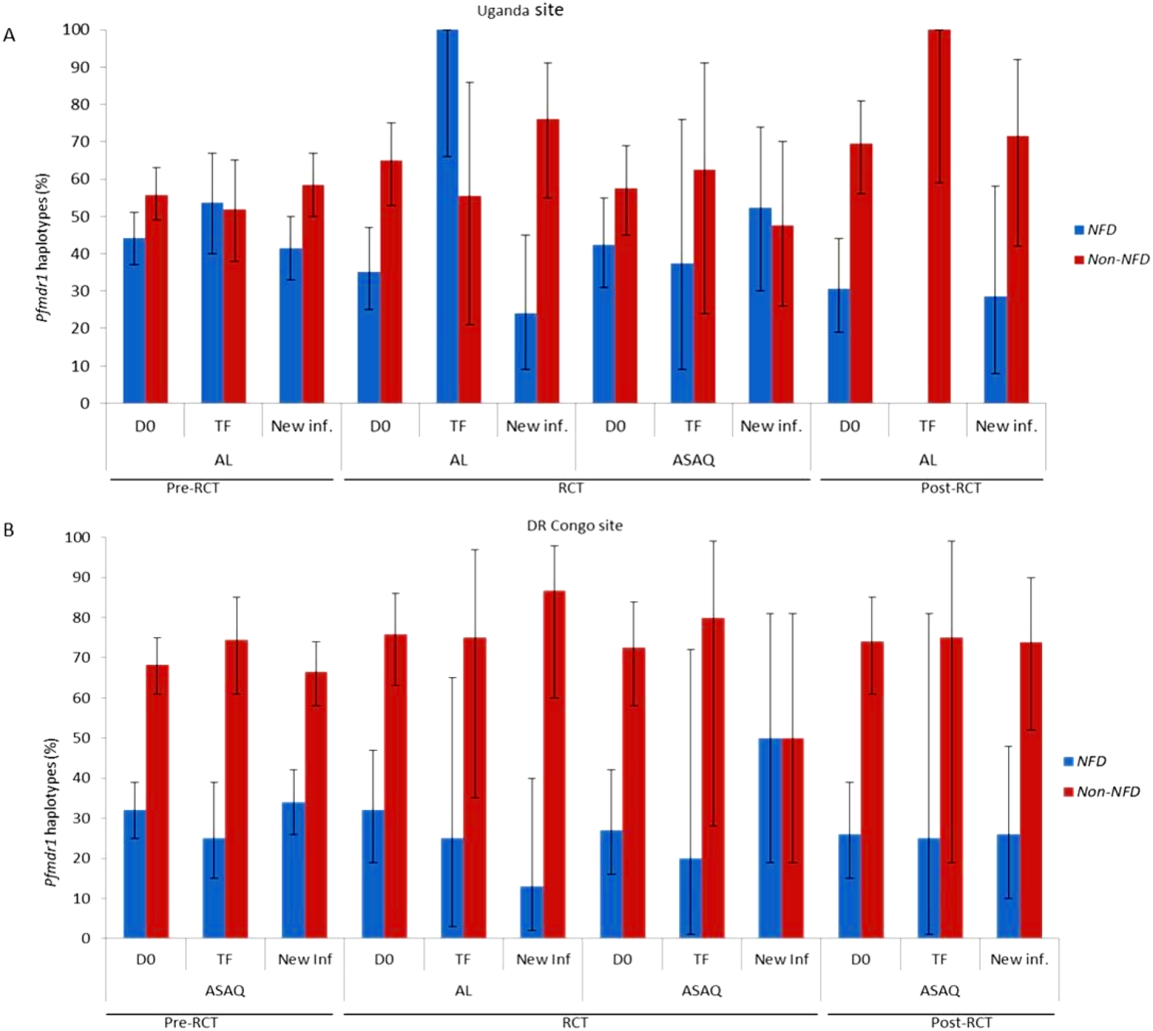
Comparison of the distribution of *Pfmdr1 NFD* and non-*NFD* haplotypes (95% Confidence intervals) by site in pre- and post-treatment outcomes in AL and ASAQ treatment arms. Haplotypes present amino acid residues at codon N86Y, Y184F, D1246Y (N = Asparagine, Y = Tyrosine, F = Phenylalanine, D = Aspartic acid, K = Lysine, T = Threonine). D0 = Day 0, TF = PCR-corrected treatment failure, ACPR = Adequate clinical and parasitological response.

Further, we assessed the selection of *Pfmdr1 NFD* haplotype (N86- 184F-D1246 *vs*. non-*NFD* haplotypes comparing baseline with post-treatment isolates (Fig 1). In the DR Congo-site, the proportion of the *Pfmdr1*-*NFD* haplotypes did not significantly change in isolates from patients that received either same ASAQ or AL as an alternative drug in the course of the study (Fig 1). However, for the Ugandan site (with exception of the RCT phase), there was a marginal reduction in the proportion of isolates harbouring *Pfmdr1*-*NFD* haplotype in new infections in AL treatment arm (p = 0.052), (Fig 1). Although not statistically significant, the general trend indicates that the proportion of the *Pfmdr1 NFD* in treatment failure (TF) slightly increased from the baseline in pre-RCT and RCT in AL retreatment arm (Fig 1A). On the contrary, retreatment with ASAQ seems to have the opposite effect on the proportion of *NFD* haplotype (Fig 1).

### Association between *Pfmdr1* alleles and the treatment failure after treatment with AL and ASAQ in randomization phase

We assessed the relative risk (RR) of treatment failure (PCR adjusted) in the RCT phase and of various *Pfmdr1* alleles and as well, the haplotypes following treatment with AL and ASAQ (Table 4). The parasites harbouring the *Pfmdr1* 86Y had significantly lower risk of treatment failure post AL treatment compared to 86N (relative risk [RR] = 0.34; 95% CI = 0.11–1.05, p = 0.04), (Table 4). The *Pfmdr1* Y184 and D1246 were not associated with risk of treatment failure after AL treatment, (RR = 0.50; 95% CI: 0.17–1.49, p = 0.26) and (RR = 1.02; 95% CI: 0.42–2.47, p = 1.0), respectively (Table 4). Likewise, the *NYY* haplotype was not significantly associated with risk of treatment failure relative to the *Pfmdr1 NFD* haplotype after exposure to AL (RR = 1.16; 95% CI: 0.70–1.92; p = 0.59). None of the *Pfmdr1* alleles was associated with ASAQ treatment failure, (Table 4). Similarly, *Pfmdr1 NYY* and *NYD* did not seem to affect the risk of ASAQ treatment failure, (RR = 0.54; 95% CI: 0.08–3.64; p = 0.65) and (RR = 1.18; 95% CI: 0.56–2.52; p = 0.78), respectively, (Table 4).

**Table 4:** Association of *Pfmdr1* alleles and haplotypes and risk of RCR-adjusted treatment failure following treatment with Artemether-Lumefantrine and Artesunate-Amodiaquine.

Treatment	*Pfmdr1* SNPs	Treatment outcomes, n (%)		RR (95%,CI)	*P-value*
		Success, n/N(%)	Failure, n/N(%)		
AL	N86	57/84(67.9)	27/84(32.1)	0.34(0.11–1.05)	**0.04**^†^
	86Y	24/27(88.9)	3/27(11.1)		
	Y184	61/87(70.1)	26/87(29.9)	0.50(0.17–1.49)	0.27^†^
	184F	17/20(85.0)	3/20(15.0)		
	D1246	69/96(71.9)	27/96(28.1)	1.02(0.42–2.47)	1.0^†^
	1246Y	10/14(71.4)	4/14(28.6)		
	*N-F-D*^¥^	20/24(83.3)	4/24(16.7)	1.71(0.39–7.48)	0.59^†^
	*N-Y-Y*	5/7(71.4)	2/7(28.6)		
	*N-Y-D*	21/29(72.4)	8/29(27.6)	1.66(0.57–4.8)	0.51^†^
	*Y-F-D*	5/6(83.3)	1/6(16.7)	1.00(0.14–7.39)	1.00^†^
	*Y-Y-D*	6/9(66.7)	3/9(33.3)	2.0(0.55–7.2)	0.36^†^
	*Y-Y-Y*	1/1(100.0)	0/1(0)	-	1.0^†^
ASAQ	N86	59/79(74.7)	20/79(25.3)	1.03(0.47–2.26)	0.94
	86Y	17/23(73.9)	6/23(26.1)		
	Y184	49/68(72.1)	19/68(27.9)	0.84(0.41–1.70)	0.63
	184F	26/34(76.5)	8/34(23.5)		
	D1246	62/86(72.1)	24/86(27.9)	0.63(0.21–1.86)	0.54^†^
	1246Y	14/17(82.4)	3/17(17.6)		
	*N-F-D*^¥^	16/23(69.6)	7/23(30.4)	0.54(0.08–3.64)	0.65^†^
	*N-Y-Y*	5/6(83.3)	1/6(16.7)		
	*N-Y-D*	23/36(63.9)	13/36(36.1)	1.18(0.56–2.52)	0.78
	*Y-F-D*	7/7(100.0)	0/7(0)	-	-
	*Y-Y-D*	7/7(100.0)	0/7(0)	-	-
	*Y-Y-Y*	0/0(0)	0/0(0)	-	-

SNP = single nucleotide polymorphisms; ACPR = adequate clinical and parasitological response, Failure = All treatment failure are PCR-adjusted, AL = Artemether-Lumefantrine; ASAQ = Artesunate-Amodiaquine; RR = Relative Risk; CI = 95% confidence interval;

^¥^ = *Pfmdr*1-*NFD* haplotypes was used as a reference for comparison.

^†^Fisher-Exact probability test with Yates-correction was used if sample size was <5.

Haplotypes present amino acid residues at codon N86Y, Y184F, D1246Y (N = Asparagine, Y = Tyrosine, F = Phenylalanine, D = Aspartic acid, K = Lysine, T = Threonine. Significant *P*-values (95% CI) are presented in boldface

### Predictors for *P*. *falciparum* recrudescence and new-infections

Anaemia was a strong predictor of recrudescence in patients treated with AL (aOR = 16.71; 95% CI: 1.86–149.26, p = 0.012), (S1 Table). There was no evidence to suggest the role of *Pfmdr1* SNPs in modulating the risk of recrudescence in this AL treatment arm (S1 Table). In addition, in the AL treatment arm, *Pfmdr1* 184F was associated with an increased risk of new-infections (aOR = 2.92; 95% CI: 0.99–8.53; p = 0.0486). None of the *Pfmdr1* variants and were associated with risk of PCR-corrected recrudescent or new infections in the ASAQ arm. Analysis of the *Pfmdr1*-*NFD* and non-*NFD* haplotypes did not reveal significant association trend with either the re-infection or recrudescence in either AL or ASAQ treatment arms, (S1 Table).

### Survival estimates to recrudescence and new infections stratified by Pfmdr1 alleles

The 28-days survival estimates of the *Pfmdr1* alleles in relation to the occurrence of recrudescence was conducted for AL (n = 110) and ASAQ (n = 104) treatment arms in the RCT phase.

**Fig 2:**
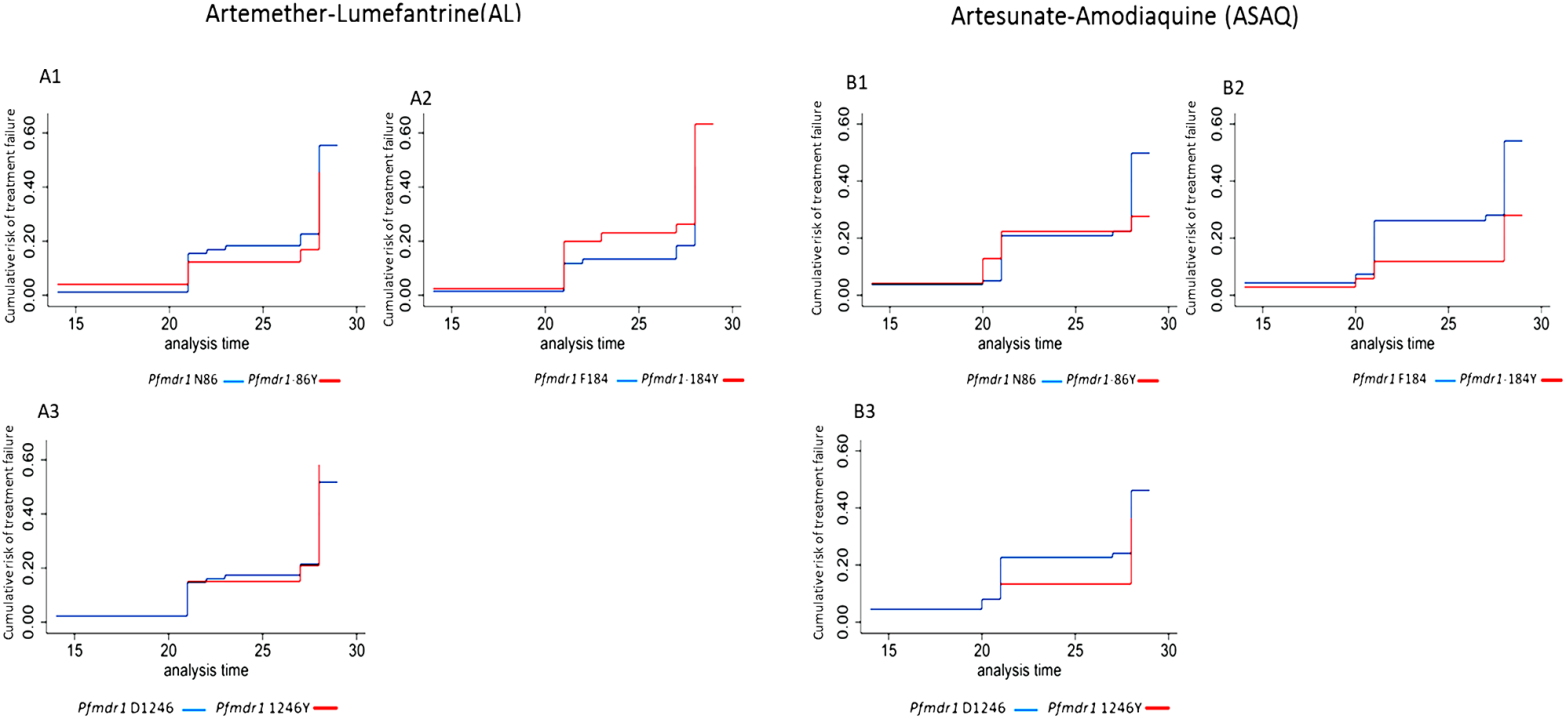
Survival estimates of the cumulative risk of treatment failure by *Pfmdr1* alleles in patients treated with AL and ASAQ in the randomisation phase. Fig 2(A1-A3) represent the risk of PCR-adjusted recrudescence in participants who received AL harbouring by *Pfmdr1* variant. Similarly, Fig 2(B1-B3) represents the risk of PCR-adjusted recrudescence in participants who received ASAQ by *Pfmdr1* variants.

We noted no difference in AL treatment arm. Time to treatment failure tended to occur late (> 20days) after AL treatment and was comparable between *Pfmdr1* N86 *vs* 86Y, (27.7 mean days (md); 95% CI: 27.4–28.0 *vs* 27.4 md; 95% CI: 26.3–28.5; p = 0.97). Similarly, the mean time to treatment failure in patient harbouring either *Pfmdr1* 184F and 184Y had comparable survival estimates [27.9md; 95% CI: 27.7–28.1 *vs* 27.3md; 95% CI: 26.4–28.1; p = 0.75). Similar trend was observed with regard to the *Pfmdr1* D1246 and 1246Y, (27.6md; 95% CI: 27.1–27.9 *vs* 28md; 95% CI: 28–28; p = 0.42), (Fig 2).

Treatment failure tended to occur late (>20 days) after ASAQ treatment arm with close similarity between *Pfmdr1* N86 *vs* 86Y alleles, (27.5md; 95% CI: 26.9–27.9 *vs* 27.3md, 95% CI: 26.4–28.2; p = 0.97). Similarly, patients harbouring either *Pfmdr1* 184F or Y184 also had comparable mean-time to recrudescence [(27.6md; 95% CI: 27.1–28.0 *vs* 27.2md; 95% CI: 26.2–28.1; p = 0.75). Furthermore, no difference was observed when comparing *Pfmdr1* D1246 and 1246Y (27.4md; 95% CI: 26.9–27.9 *vs* 27.5md; 95% CI: 26.6–28.4; p = 0.42), (Fig 2). A similar trend was observed when considering new-infections (PCR-adjusted) and crude treatment failure (PCR-unadjusted) in both AL and ASAQ treatment arms, (S1 Fig).

## Discussion

Our data suggest a limited drug-mediated selection of *Pfmdr1* alleles after exposure to AL and ASAQ suggesting a restricted impact on the selection of *Pfmdr1* alleles previously associated with reduced sensitivity to 4-aminoquinolines and aryl-aminoquinolines partner drugs. The results supplement the evidence on the ACTs as a possible rescue treatment for curing recurrent *P*.*falciparum* malaria with restricted impact on the selection of drug-resistant malaria parasites [27].

We observed significant variation in the baseline *Pfmdr1* among the two sites. In Uganda, the baseline prevalence of the *Pfmdr1* N86 was significantly higher while in DR Congo *Pfmdr1* 86Y was more predominant. However, on both sites, there was no evidence of drug-mediated selection in either phase except the observed borderline directional selection for *Pfmdr1* 184F and borderline significance for *Pfmdr1* N86 in recurrent infections after AL treatment (Table 3). The observed selection could indicate that AL for the *Pfmdr1* 184F allele in patients with malaria recurrences and could subsequently increase the circulation of parasite harbouring the *Pfmdr1 NFD* haplotype known to tolerate high concentrations of lumefantrine [24]. The results mirror the previous finding that demonstrated a strong selection of *Pfmdr1* 184F that persisted for a prolonged duration (2months) after treatment with AL, but not ASAQ in Uganda [30]. Reports in DR Congo suggests a high prevalence of *Pfmdr1* 86Y, 184F, D1246 [31] while in Uganda evidence suggest a high prevalence of *Pfmdr1* N86 in most settings[17,30]. However, we found no marked difference in the frequency of *Pfmdr1 NFD* and *non-NFD* haplotypes in Ugandan and DR Congo in patients. Although ASAQ is the recommended first-line treatment in DR Congo, both AL and ASAQ are used in the treatment of uncomplicated malaria exerting a balanced selection pressure on *Pfmdr1* alleles compared to Uganda where AL is more widely used. The trend suggests that usage of different or alternating ACTs may delay the development of resistance by exerting inverse selection pressure on the parasites [26]. We found significant evidence increased in relative risk of treatment failure (PCR-adjusted) associated with *Pfmdr1*-N86 in patients treated with AL, however, none of the *Pfmdr1* alleles was associated with ASAQ treatment failure, (Table 2). The findings are in concordance with a meta-analysis data that suggested 5-folds increased the risk of treatment failure in the patients treated with AL[26]. However, we could not associate the increased risk of ASAQ of failure in patients harbouring *Pfmdr1* 86Y after treatment which compares to other reports elsewhere [16,26]. None of the *Pfmdr1* haplotypes was associated with AL or ASAQ treatment, however, there were few *Pfmdr1* haplotypes detected in post-treatment to allow meaningful statistical power for comparison.

In multivariate analysis, none of the *Pfmdr1* alleles had no significant association with treatment failure or risk of new-infections after treatment with ASAQ. In AL treatment arm, patients harbouring *Pfmdr1*-184F had significantly higher risk of new-infection, in line with a study that reported an association between lumefantrine and *Pfmdr1* F184 in *ex-vivo* assay [32]. Interestingly, patients with anemia were increasingly at high risk of recrudescence in AL treatment arm, this may be supported the fact that the risk of acquiring anaemia is high in patients with high parasite density (<200,000/μL)) which was shown to be an independent risk factor for clinical treatment failure by up to 60% for the different antimalarial treatment[33]. This is further reinforced by the evidence that *falciparum* infected patients that responded poorly to the treatment were more likely to recrudesce and consequently increased their risk of anaemia [34]. However, it is reassuring that, with the high efficacy of AL, the risk of treatment failure and selection of drug-resistant parasites after repeated ACT administration is low in most endemic settings in SSA. Additionally, *Pfmdr1 NFD* haplotype showed increasing trend in the RCT phase in patients with TF administered AL as a rescue treatment, these findings are in line with evidence from previous studies that demonstrated increased level after AL exposure [13,16]. The comparison of the pre- and post-treatment indicate an increasing overall trend indicate that the proportion of the *Pfmdr1 NFD* in treatment failure(TF) slightly increased from the baseline in pre-RCT and RCT in AL retreatment arm (Fig 1), however, this was not statically significant. On the contrary, retreatment with ASAQ seems to have the opposite trend on the proportion of *Pfmdr1 NFD* haplotype (Fig 1). In most of the countries where AL is the first-line treatment, the proportion of the *Pfmdr1* Y86 seems to decline over time decreasing the overall proportion of *NFD* haplotypes, further depicting the opposing drug-mediated selection of resistant alleles [29,35,36].

With regard to the impact of treatment and re-treatment on selection for drug resistance markers, we did not observe a directional selection of *Pfmdr1* 86Y, Y184 and 1246Y in patients that received ASAQ as their first-line and as an alternative therapy in RCT phase in both sites. Similarly, the same trend for with regard to the selection of the *Pfmdr1* N86 and D1246 in patients that received AL as the first-line regimen and then randomised to receive the same AL or ASAQ as a rescue therapy in RCT phase. Generally, there is evidence that (re-) treatment with same or alternative ACT offers limited impact in terms of selection for *Pfmdr1* resistant alleles and haplotypes as documented in most of the trial phases, pre-RCT, RCT, and post-RCT. This could be further be supported by other studies that documented related fitness cost as it was shown by Fröberg *et al* [37], that demonstrated that although ASAQ selects for *Pfmdr1* mutations associated with ASAQ reduced susceptibility, there is a significant fitness-cost incurred to the parasite rendering them unfit to compete with the wild type variants. Our findings, thus, suggest the limited impact of the ASAQ and AL on selection for *Pfmdr1* allele when used as a rescue therapy in (re-)treatment of recurrent infections in real-life settings.

Currently, there are limited systematic studies that evaluated changes of *Pfmdr1* alleles and haplotypes in the context of (re-)treatment approach. In a prolonged follow-up in Uganda, there was a clear significant selection of *Pfmdr1* N86, 184F and D1246 alleles prior treatment with AL but not after ASAQ treatment [30]. Also, recent findings in Burkina Faso presented conflicting results on select of *Pfmdr1* N86 [38,39]. Other studies suggest increased selection of the *Pfmdr1* alleles prevalence in the parasite population following treatment with AL and ASAQ [14,18,26,40,41]. The plausible explanation for the observed differences could be the differences in country treatment policy. Also, the shorter half-life of lumefantrine is unlikely to have conferred strong directional selection for *Pfmdr1* copy number in sub-Saharan African parasite populations that are known to harbour less copy number compared to SEA where MQ is widely used [12,26]. I addition, the synergistic action of artemether and lumefantrine exerted on the parasite could also play a role, artemether has retained the high efficacy due to the rapid reduction in parasite biomass rendering a relatively low risk to parasite infections being exposed to the selective pressure of the partner's drug.

Furthermore, we presume that other factors such as drug bioavailability, transmission intensity, intrinsic human genetic factors and immunity could also contribute to the observed outcomes, however, these were not explored in the scope of the present study.

As a limitation to the present study, the association between risks of treatment failure in relation to *Pfmdr1* genotypes was restricted by the low sample size in the RCT and post-RCT. Furthermore, we did not analyse changes in *Pfmdr1* copy number variation (CNV) that has been previously associated with lumefantrine tolerance. In GMS where parasites are resistant to artemisinin derivatives and partner drugs such as Mefloquine (MQ), increased *Pfmdr1* CNVs is reportedly high in contrast to most endemic settings in sub-Saharan Africa [12,26]. Nonetheless, despite the observed occurrence of wild haplotype *Pfmdr1* N86, Y184, and D1246 alleles in pre- and post-treatment isolates, AL still maintain high 28-day *in vivo* efficacy (>90% PCR-adjusted efficacy rate) in parasite populations in Uganda and elsewhere in sub-Saharan Africa [8]. In DR Congo, ASAQ retains high sensitivity due to the demonstrated low occurrence of the *Pfmdr1 YYY* and also the rare occurrence of SVMNT haplotype reported elsewhere[42]. Overall, there is limited reports evidence of declining ACT efficacy, except the demonstrated marginal elongation of parasites clearance time (PCT) and cases of treatment failure in clinical the coast of Kenya and report of imported malaria cases isolated from UK based travellers returning from Africa [43,44]. The high efficacy is depicted in the recent larger multicentre studies that demonstrated distinct clusters of parasite population in Africa and South-East Asian isolates with the limited dispersal of parasites strains carrying *Pfkelch*-13 key mutations in Africa settings and these include baseline samples from our QuinACT trial[45–47]. However, despite these observations, new-infections remain exposed to sub-therapeutic levels of partner’s drugs due to their relative longer elimination half-lives and this may increase drug-mediated selective pressure on the *Pfmdr1* gene. Additionally, over-prescription and over-the-counter prescription of ACTs both first-line and the second line should be restricted to reduce unnecessary drug pressure.

Furthermore, our data suggest no difference in time to PCR-adjusted treatment failure in both treatment arms in standard 28-days follow-up during the randomisation phase (Fig 2, and S1 Fig). Also, similar results were observed when considering crude treatment failure (PCR unadjusted treatment failure) and PCR-corrected new-infections. There was a low risk of early PCR-adjusted treatment failure supporting the previous reports that the risk of *Pfmdr1* associated new-infections occurs later in >20days [26]. In most settings in Africa, the majority of malaria recurrences are classified as new-infections after PCR-correction. However, we did not observe significant differences in survival estimates between *Pfmdr1* wild variants treatment failure or new-infections in both AL and ASAQ arms as reported elsewhere[26].

In light of our findings, we have demonstrated that AL and ASAQ can be used as a rescue treatment to treat recurrent falciparum malaria in real-life settings without exerting additional selection pressure on the *Pfmdr1* gene. The observed selection of *Pfmdr1* 184F in new infections after treatment with AL warrants further investigations as the drug is increasingly used for (re-)treatment of recurrent malaria infections in several endemic countries. It may seem, therefore, plausible to consider the use of alternative ACT with the opposing mechanism of selection against on the *Pfmdr1* gene as a stopgap strategy to counterbalance drug-mediated should ACT resistance worsen due to selection pressure. For instance, in countries where treatment policy recommends AL as the first-line therapy, ASAQ or DHA-PQ could be considered reciprocally to reduce the risk of directional drug-mediated selection of resistant alleles, a similar strategy has been previously supported [26,37,48]. Additionally, there is accrued evidence that simultaneous use of multiple first-line ACT therapies (MFT) is an ideal strategy to delay evolution and spread of resistance against artemisinin derivatives and partners drugs, on-going evaluation will inform on the appropriate models to facilitate the implementation and management of MFT strategy for the treatment of uncomplicated malaria[49–51]. Thus, to counterbalance the effect of drug-mediated selection, deployment of alternative ACTs with antagonistic effect or implementation of strategies like MFT may offer a way to mitigate resistance against partner drugs. Indeed, a recent clinical trial in Uganda demonstrated the superiority of ASAQ compared to AL in reducing the risk of recurrences, and this was partly attributed to the parasite genetic background [52]. In the meantime, however, using the same ACT as rescue therapy does not seem to compromise treatment outcomes, presumably due to high efficacy of artemisinin derivatives and the fact that majority of malaria recurrences due to new infections. Ensuring patients adherence to (re-)treatment with ACT regimens and providing accurate malaria diagnosis before antimalarial treatment will undoubtedly contribute the most to enhancing the continued efficacy of the drugs.

Of note, *de facto* ACT resistance is yet to spread or emerge in most endemic settings in Africa, however, future antimalarial drug discovery and clinical trials need to continue evaluation of new treatment combination with opposing mechanisms of selection to reduce the potential risk for selection in malaria endemic transmission settings where patients are exposed to frequent treatment and re-treatment with ACTs. Nonetheless, the present study provides timely and encouraging molecular results on the impact of drug-mediated selection to support the use the same or alternative ACTs for (re-)treatment in endemic countries. In conjunction with these strategies, regular monitoring is warranted to provide evidence to inform policy on the long-term effectiveness of ACT in control and elimination efforts.

## Conclusion

In summary, our study demonstrates the limited impact of (re-)treatment with AL or ASAQ on selection for *Pfmdr1* alleles and haplotypes. These findings further supplement the evidence use of same or alternative ACTs as a rescue therapy for (re-)treatment of recurrent *P*.*falciparum* infections. Continued monitoring of genetic signatures of resistance is warranted to timely inform malaria (re-)treatment policies and guidelines.

## Supporting information

S1 Table Multivariate logistic regression model of the association between *Pfmdr1* SNPs and PCR-corrected recrudescence and new infections of patients treated with AL and ASAQ on day 28 follow-up in RCT phase. (DOCX)

S1 Fig Cumulative risk of crude treatment failure (PCR-unadjusted) by *Pfmdr1* in patients treated with AL and ASAQ in the randomisation phase. Figure A1-A3 represent the risk of crude treatment failure in patents who received AL by *Pfmdr1* variants. Figure B1-B3 represents the risk of crude treatment failure in participants who received ASAQ by *Pfmdr1* variants.
(TIF)
