# Supplementary material for: Impact of treatment and re-treatment with artemether-lumefantrine and artesunate-amodiaquine on selection of Plasmodium falciparum multidrug resistance gene-1 polymorphisms in the Democratic Republic of Congo and Uganda
Source: PLoS One. 2018 Feb 1;13(2):e0191922. doi: 10.1371/journal.pone.0191922 (PMC5794077; doi:10.1371/journal.pone.0191922)
Supplement: S1 Table — (DOCX) [file pone.0191922.s001.docx]

**S1 Table.** Multivariate logistic regression model of the association between *Pfmdr1* SNPs and PCR-corrected recrudescence and new infections of patients treated with AL and ASAQ on day 28 follow-up in RCT phase.

|  |  |  |  |  |  |  |  |  |  |  |
| --- | --- | --- | --- | --- | --- | --- | --- | --- | --- | --- |
| **Characteristics** | | **Treatment randomisation** | | | | | | | |  |
|  |  | **Artemether-Lumefantrine(AL)** | | | |  | **Artesunate-Amodiaquine(ASAQ)** | | | |
|  |  | **Recrudescent infections** | | **New infections** | |  | **Recrudescent infections** | | **New infections** | |
|  |  | **aOR (95%, CI)** | ***P-value*** | **(95%, CI)** | ***P-value*** |  | **aOR (95%, CI)** | ***P-value*** | **aOR (95%, CI)** | ***P-value*** |
| Site |  |  |  |  |  |  |  |  |  |  |
|  | DR Congo | 1 |  | 1 |  |  | 1 |  | 1 |  |
|  | Uganda | 2.03(0.23-17.93) | 0.52 | 2.79 (0.85-10.42) | 0.09 |  | 0.94 (0.17 – 5.23) | 0.95 | 0.92(0.24-3.52) | 0.91 |
| Age |  |  |  |  |  |  |  |  |  |  |
|  | >2years | 1 |  | 1 |  |  | 1 |  | 1 |  |
|  | <2years | 0.23(0.04-1.44) | 0.12 | 0.49(0.15-1.63) | 0.24 |  | 0.66(0.13 – 3.37) | 0.62 | 0.75(0.22-2.64) | 0.67 |
| Anaemia |  |  |  |  |  |  |  |  |  |  |
|  | No |  |  | 1 |  |  |  |  |  |  |
|  | Yes | 16.71(1.86-149.26) | **0.012** | 1.51(0.22-10.45) | 0.66 |  | † |  | 2.65(0.44-16.01) | 0.29 |
| Fever (37.5 C) |  |  |  |  |  |  |  |  |  |  |
|  | No | 1 |  | 1 |  |  | 1 |  | 1 |  |
|  | Yes | 1.67(0.22 – 12.62) | 0.62 | 0.55(0.18-1.64) | 0.28 |  | 0.46 (0.10 – 2.04) | 0.31 | 0.81(0.25-2.62) | 0.72 |
| Gmpd |  |  |  |  |  |  |  |  |  |  |
|  | No | 1 |  | 1 |  |  | 1 |  |  |  |
|  | Yes | 3.31(0.32-34.25) | 0.32 | 0.99(0.31-2.90) | 0.94 |  | 1.38 (0.29 – 6.51) | 0.68 | 0.65(0.21-1.97) | 0.45 |
| *Pfmdr1* c86 |  |  |  |  |  |  |  |  |  |  |
|  | N86(Wt) | 1 |  | 1 |  |  | 1 |  |  |  |
|  | 86Y(Mut) | 2.61(0.28-24.77) | 0.40 | 1.51(0.37-6.13) | 0.56 |  | 0.92(0.13-6.59) | 0.94 | 0.28(0.04-1.79) | 0.18 |
| *Pfmdr1* c184 |  |  |  |  |  |  |  |  |  |  |
|  | Y184(Wt) | 1 |  | 1 |  |  | 1 |  |  |  |
|  | 184F(Mut) | 1.71(0.32-8.61) | 0.52 | 2.92(0.99-8.53) | **0.05** |  | 0.88(0.18 -4.21) | 0.88 | 0.29(0.08-10.38) | 0.06 |
| *Pfmdr1* c1246 |  |  |  |  |  |  |  |  |  |  |
|  | D1246(Wt) | 1 |  | 1 |  |  | 1 |  |  |  |
|  | 1246Y(Mut) | 2.44(0.24-23.91) | 0.44 | 2.05(0.53-7.96) | 0.35 |  | 0.76 (0.08-7.69) | 0.82 | 1.65(0.38-7.13) | 0.50 |
|  |  |  |  |  |  |  |  |  |  |  |

aOR=Adjusted odd ratio; Gmpd=Geometric mean parasite density; Wt=Wildtype; Mut=Mutant ; NA=Not applicable; †=Variables was omitted because of collinearity. Significant *P*-values at 95% CI are presented in boldface;
