# Supplementary figures and images for: Impact of treatment and re-treatment with artemether-lumefantrine and artesunate-amodiaquine on selection of Plasmodium falciparum multidrug resistance gene-1 polymorphisms in the Democratic Republic of Congo and Uganda
Source: PLoS One. 2018 Feb 1;13(2):e0191922. doi: 10.1371/journal.pone.0191922 (PMC5794077; doi:10.1371/journal.pone.0191922)

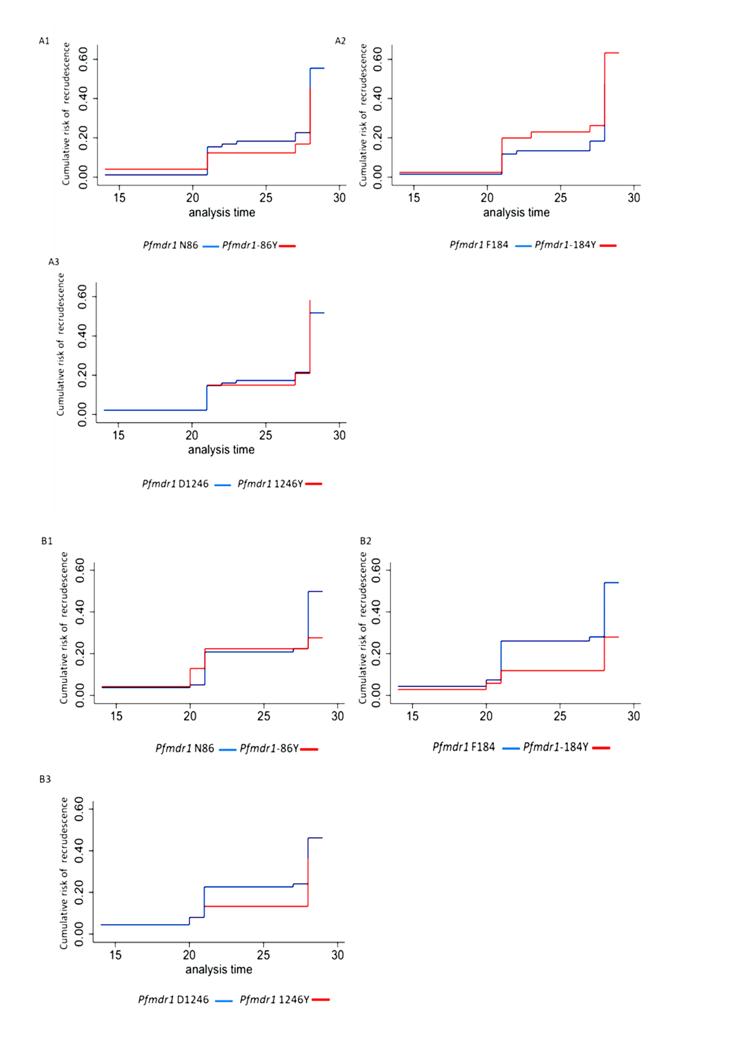

Supplement: S1 Fig — Figure A1-A3 represent the risk of crude treatment failure in patents who received AL by Pfmdr1 variants. Figure B1-B3 represents the risk of crude treatment failure in participants who received ASAQ by Pfmdr1 variants. (TIF) [file pone.0191922.s002.tif]
